# An Examination of Psychomotor Disturbance in Current and Remitted MDD: An RDoC Study

**DOI:** 10.20900/jpbs.20200007

**Published:** 2020-04-17

**Authors:** Stewart A. Shankman, Vijay A Mittal, Sebastian Walther

**Affiliations:** 1 Department of Psychiatry and Behavioral Sciences, Northwestern University, Chicago, 60611, USA; 2 Department of Psychology, Northwestern University, Evanston, 60208, USA; 3 Translational Research Center, University Hospital of Psychiatry, University of Bern, Bern, 3008, Switzerland

**Keywords:** psychomotor agitation, psychomotor retardation, major depressive disorder, remitted depression

## Abstract

Major depressive disorder (MDD) is a serious public health problem that has, at best, modest treatment response—potentially due to its heterogeneous clinical presentation. One way to parse the heterogeneity is to investigate the role of particular features of MDD, an endeavor that can also help identify novel and focal targets for treatment and prevention efforts. Our R01 focuses on the feature of psychomotor disturbance (e.g., psychomotor agitation (PmA) and retardation (PmR)), a particularly pernicious feature of MDD, that has not been examined extensively in MDD. Aim 1 is comparing three groups of individuals—those with current MDD (*n* = 100), remitted MDD (*n* = 100), and controls (*n* = 50)—on multiple measures of PmR and PmA (assessed both in the lab and in the subjects’ natural environment). Aim 2 is examining the structural (diffusion MRI) and functional (resting state fMRI) connectivity of motor circuitry of the three groups as well as the relation between motor circuitry and the proposed indicators of PmR and PmA. Aim 3 is following up with subjects three times over 18 months to evaluate whether motor symptoms change in tandem with overall depressive symptoms and functioning over time and/or whether baseline PmR/PmA predicts course of depression and functioning. Aim 3 is particularly clinically significant. Finding that motor functioning and overall depression severity co-vary over time, or that motor variables predict subsequent change in overall depression severity, would support the potential clinical utility of these novel, reliable, and easily administered motor assessments.

## INTRODUCTION

### Public Health Significance of Examining PmA and PmR

Major depressive disorder (MDD) is one of the leading causes of disability in the US and is associated with significant economic consequences [1–3]. Although treatments have been developed, response rates are modest (~40–50%) [4,5].One reason for this mixed efficacy is that MDD has different clinical profiles and, therefore, likely has different pathophysiologies. Indeed, there are over 1000 different combinations of symptoms that could lead to an MDD diagnosis [6].

One way to parse the heterogeneity is to investigate the role of particular features of MDD, an endeavor that can also help identify novel and focal targets for treatment and prevention efforts. One of the most pernicious symptom clusters in MDD is psychomotor disturbance. Psychomotor disturbance is typically classified as either psychomotor retardation (PmR, i.e., a slowing or reduction in physical movements) or psychomotor agitation (PmA, i.e., an increase in purposeless and often unintentional motor activity) [7]. Both symptom clusters have consistently been associated with severe forms of MDD (and perhaps a qualitatively different subtype) [8,9] and worse treatment response in multiple trials [9–11]. PmR and PmA are not specific to MDD as they occur in numerous other disorders (e.g., schizophrenia, Parkinson’s disease). Notably, given the importance of PmR and PmA to psychopathology, NIMH recently added psychomotor abnormalities as a sixth domain of constructs to their Research Domain Criteria (RDoC) matrix [12,13].

While psychomotor disturbance has been acknowledged as an important component of depression for decades, its measurement and conceptualization has been extremely coarse. Traditionally, psychomotor disturbance has only been assessed by patient self-report or an observer’s global observations. These conceptualizations are problematic because they (a) confound the cognitive concomitants of psychomotor disturbance (e.g., poor concentration, fatigue) with motoric ones, (b) do not separate the different components of psychomotor disturbance (e.g., motor initiation vs motor inhibition), and (c) are influenced by patient/observer’s reporting biases. They also do not allow for patients to have both PmR and PmA, which, although rare, does occur [8,14,15].

To overcome these problems, our recently funded R01 seeks to examine psychomotor disturbance across several units of analysis, including self-report, observation, laboratory assessment, and naturalistic behavior (outside the lab). Each approach has its own unique benefits and provides distinct information. For example, the study includes laboratory assessments which measure motor disturbance using more fine-grained methods that are objective (and thus not subject to a patient or observer’s reporting bias) and shown to be reliable. One laboratory task asks subjects to apply constant pressure on a strain gauge for a period of time. The variability in the subject’s ability to maintain that pressure (and thus limit irregular muscle contractions) is called *Force Variability*. Another laboratory task has subjects draw patterns on a tablet computer. One metric extracted from this task is called *Velocity Scaling*, which reflects the ability to increase pen movement velocity across shorter and longer target distances. Numerous studies on disorders such as Parkinson’s and schizophrenia have used Force Variability and Velocity Scaling as indicators of PmA and PmR, respectively [16,17]. However, few depression studies (e.g., ref. [18]) have used them, despite the importance of PmA and PmR to mood disorders. Thus, the first goal of this study is to characterize the PmR and PmA deficits in depression using a range of assessment modalities, including state-of-the-art assessments of motor behavior, such as Force Variability (for PmA) and Velocity Scaling (for PmR).

### Psychomotor Disturbance Assessed Outside of Lab

PmR and PmA will also be assessed in daily behaviors measured outside of the lab. This is a significant feature of the study as it provides objective, continuous assessments of motor behaviors in naturalistic settings. We are employing two “outside of lab” measures. First, we are capturing naturalistic gross motor behavior using wrist-worn actigraphy. Actigraphy is a non-invasive method of continuously recording total body movement over days and weeks, and has been used to reliably assess (a) activity level and (b) stability of movement patterns (our indicators of PmR and PmA, respectively), even in severe psychopathologies [19]. Importantly, MPI Walther has shown that actigraphy-assessed motor behavior predicts the course of MDD over and above clinician ratings of PmR and PmA (see PRELIMINARY STUDIES below).

Second, we are assessing PmR and PmA through subjects’ naturalistic **typing behavior on their smartphones**. Smartphones are near ubiquitous (e.g., in the age range of this study, ownership rates are ~92%) [20], and thus offer an excellent platform on which to study naturalistic behavior [21–23]. Towards this aim, Co-I Leow developed a smartphone app (*BiAffect)* that uses the phone’s passive sensors to unobtrusively assess typing behavior as people type their normal texts, social media posts, etc. One indicator of typing behavior, interkey delay (i.e., time between two consecutive key presses), is especially likely to relate to psychomotor disturbance. Specifically, longer interkey delays (i.e., slower typing speed) is likely to be an indicator of PmR, and variability in typing speed is likely to be an indicator of PmA. Indeed, Co-I Leow showed that keystroke behavior (a) correlates with other measures of motor processing speed (i.e., Trail making Test A) and (b) over and above mood symptoms, prospectively predicts depressive symptoms 8 weeks later [24] (see PRELIMINARY STUDIES below).

### Possible Neural Mechanisms of PmA and PmR

The neural basis of psychomotor disturbances in depression is largely unknown. A small handful of studies have found that PmR is associated with altered perfusion in particular cortical and subcortical areas [25], alterations of white matter motor pathways [26,27], and hypodopaminergic states [28,29]. Rather than examining whether neurotransmitters or isolated neural structures are associated with PmA and PmR, a more informative and parsimonious approach is to examine whether large scale neural circuits are simultaneously involved in PmA and PmR. Decades of animal and human work have identified three separate circuits that mediate different aspects of basic motor behavior—basal ganglia circuitry, cerebellar-thalamo-cortical circuitry, and cortico-cortical circuitry [13]. Numerous studies have used this neural framework to examine motor abnormalities in diseases such as schizophrenia, Parkinson’s disease, and bipolar disorder. There is therefore a **strong scientific premise** for examining the role that these motor circuits play in PmA and PmR in MDD. To date, studies of neural networks in unipolar depression have focused on networks such as the default mode network [30] and frontoparietal control networks [31], and not on motor networks (however, studies in bipolar disorder have examined these circuits [32,33] and suggest that they may be abnormal in unipolar depression). The study will therefore provide novel insights into neural mechanisms that are involved in motor disturbances in MDD [34].

As noted above, the specific motor circuits that are involved in PmA and PmR in MDD are unclear. However, several tentative hypotheses can be made. The cortico-cortical network (e.g., inputs from DLPFC or dorsal ACC to preSMA and SMA) is likely to be hypoactive in PmR given this network’s role in movement initiation and action selection. In contrast, PmA is likely to be associated with hyperactivity in cortico-basal ganglia circuits. However, it is also possible that PmA may involve interactions between the three circuits (with particularly important contributions from the basal ganglia, cerebellum, dorsal ACC, and preSMA/SMA) as PmA likely results from multiple deficits, such as movement inhibition, timing, and termination. To test these hypotheses, we will evaluate structural and functional connectivity of motor networks in different stages of illness (i.e., in episode vs remission). As this is a new area with limited existing data to guide firm hypotheses, we will utilize a conservative analytic approach by evaluating nodes comprising the three primary networks responsible for movement and identify patterns consistent with the tentative hypotheses noted above. This approach will also allow us to identify unexpected, novel, and potentially path-breaking associations regarding motor network circuitry in PmR and PmA. The analysis plan is well suited to power this unique and innovative approach. Specifically, we will examine key motor ROIs across each of the networks, map white matter tracts comprising these networks, and aim to determine coherence within and across the motor networks (evaluating coupling between pairs of ROIs, as well as within the broader context of the complex networks). The goals of this aim are to gain new insight into brain networks underlying PmA and PmR in MDD, determine unique and overlapping pathology underlying both PmR and PmA, and determine if the phase of illness influences patterns of aberrant brain connectivity and respective motor behaviors. This will ultimately lay the groundwork for novel biomarkers and individualized interventions for this difficult to treat feature of MDD.

### The Importance of Studying Remission

The few studies that have examined motor disturbance in depression only included currently symptomatic individuals. This is problematic as these studies cannot differentiate whether the deficits are (a) a function of current symptomatology or (b) a core underlying etiological mechanism of depression. This study is therefore examining individuals currently in an episode of MDD (i.e., acute depression), as well as individuals remitted from MDD. While studies of remitted MDD cannot disentangle whether the deficit reflects a pre-morbid process or a scar of depression, they do provide evidence that the deficit is independent of symptomatology that persists into remission. Studying deficits in remitted individuals also yields strong preliminary data for longitudinal high risk studies.

### The Longitudinal Course of Motor Behavior

Previous studies have also not examined the longitudinal course of motor behaviors in the context of depression. This hole in the literature has important public health significance as it is unclear whether motor disturbance ‘tracks’ with fluctuations in depression severity over time, or whether it is independent (suggesting that motor disturbance should be treated separately from overall depression severity) [35]. To address this question, we are re-assessing the in-lab (e.g., self-report, observer assessment, Force Variability, and Velocity Scaling) and out-of-lab (e.g., actigraphy and smartphone app) motor measures and depressive symptomatology three more times during an 18-month follow-up period. This prospective, longitudinal design will allow us to not only test whether changes in state depression relate to changes in PmR and PmA over time, but also whether baseline measures of PmR and PmA (including the neuroimaging measures of motor circuitry) predict a worse course of illness. The prospective design can also address whether PmR and PmA have *independent* predictive validity on the course of depression. This is a critical question because, while one might view PmR and PmA as opposite sides of the same coin, several self-report studies have found them to be separable, with some depressed individuals exhibiting both PmR and PmA [14,15] (mirroring results seen in schizophrenia [36]). Knowing whether PmR and PmA reflect correlated vs independent mechanisms will also aid in the identification of novel (and individualized) biomarkers.

In addition to depressive symptoms as outcomes, the present study is also testing whether motor disturbance predicts functional impairment (specifically, social and occupational functioning using the SOFAS) [37] and overall quality of life (using the WHO-QOL) [38]. Our group and others have shown that motor disturbance predicts social and role functioning outcomes in other clinical populations (e.g., psychosis risk, psychosis) [39,40], but this is the first study examining these associations in depression.

Lastly, having multiple assessments of depression and motor behaviors will allow us to use lagged analyses (e.g., whether PmR at one point predict depression at subsequent time points) to explore whether PmR and/or PmA predict relapse (in the remitted MDD group) or remission (in the current MDD group). As preliminary support for this approach, Dr. Walther and colleagues recently demonstrated that observer ratings of PmR predicted response to electroconvulsive therapy [41].

### Incremental Validity over Self- or Observer-Reported PmR and PmA

For each of the aims of the grant (cross-sectional group effects, neural mechanisms, longitudinal course), we will also test for effects over and above self- or observer-reported PmR and PmA. This is an important goal because if the lab-based (e.g., Force Variability) and ecologically valid (e.g., smartphone typing) measures of motor behavior do not demonstrate incremental validity over and above self- and observer-reported PmR and PmA in predicting variables such as longitudinal course (Aim 3), their potential utility in the clinic would be diminished. Alternatively, self- and observer-reported PmR and PmA may provide complementary information to the other measures, thus yielding a complete picture of the different components of motor disturbance (a goal consistent with RDoC’s emphasis on multiple “units of analysis” (i.e., measures) of a particular construct) [42].

### Sex Differences in Motor Disturbance

A final goal of our study is to examine sex differences in motor disturbance. While it is well-established that women are at greater risk for and exhibit a different course of MDD than men [43], studies have been equivocal as to sex differences in psychomotor disturbance, with some reporting higher rates of motor disturbance in depressed men [7] and others reporting higher rates in women [44]. Given that these studies only used self- or observer-reported measures of motor disturbance, the present study is the first to test this question with more fine-grained assessments of PmR and PmA (and to test whether sex moderates the neural mechanisms and longitudinal course). This is consistent with NIH’s goal of examining sex as a biological variable. While our study is not fully powered to detect sex differences, it will provide important preliminary data regarding sex differences in psychomotor disturbance in MDD.

### Summary of Scientific and Clinical Significance

Depression is a highly prevalent disorder and, since its inception, motor slowing and/or agitated movements has been identified as one of its most pernicious features. Despite this prominence, we know startlingly little about these behaviors. Specifically, it is unclear how to best characterize PmR and PmA in individuals in episode, and whether they continue into remission. One possibility is that they remain present, but become attenuated in remission (something that our **Aim 1** will be able to test). These results would also help inform risk models of MDD. Further, little is known about the neurological basis of these movement abnormalities (something we found particularly surprising as we planned this study), including whether PmR and PmA have separate pathophysiologies. We therefore based our tentative hypotheses on what is known from other disorders and from our preliminary studies (see Brain Correlates of Actigraphy below), and then set a highly conservative analytic plan of examining the structure and function of three primary circuits that govern motor behaviors in general (**Aim 2**). As the motor networks have not been evaluated in depression, this comprehensive approach is likely to elucidate novel information about the pathogenesis of the disorder.

What is also striking is the lack of understanding about how motor behaviors change across the course of MDD. No study has examined if motor disturbance changes as overall depression and functioning improves, or if they change as remitted patients move towards relapse (questions that **Aim 3** will address). Studies of psychomotor disturbance in Parkinson’s disease and psychosis offer tremendous promise for how subtle motor biomarkers can predict clinical changes [45,46]. Finding that motor and depressive symptom changes co-vary over time, or that motor variables predict subsequent change in overall depression severity, would support the potential clinical utility of our novel, reliable, and easily administered motor assessments. For example, demonstrating that smartphone typing behavior tracks with overall depression changes would inform a future study on the feasibility and utility of updating clinicians in real time about the status of their patients (e.g., an automatic email sent from the smartphone app) [47].

## INNOVATION

Although motor dysfunction has long been viewed as a key feature of depressive disorders [7], there is a surprising lack of understanding surrounding these core behaviors. There are numerous ways that this study addresses the significant holes in this literature in innovative ways, using cutting edge methodologies.

First and foremost, the current study seeks to understand PmR and PmA using in-depth and novel measures of motor disturbance. While prior studies of motor disturbance in MDD almost exclusively utilized self- and observer-ratings, the present study is measuring PmR and PmA using multiple methods, including laboratory (e.g., force variability, velocity scaling) and ecologically valid (e.g., actigraphy, typing behavior on smartphones) methods. These sensitive measures will elucidate subtler and more fine-grained indicators of PmR and PmA (in contrast to self- and observer-reports, that largely identify more overt signs) and allow for the parameterization of motor behaviors using objective, unbiased methods.

Second, the evaluation of both structural and functional connectivity in networks responsible for motor behavior has never been attempted in studies of MDD. The well-supported models of brain networks underlying motor behavior in general will serve as an anchor point for mapping out neural networks involved in PmR and PmA. This approach has been fruitful in psychosis risk populations (providing insights into disease progression [48]), suggesting that taking a similar approach in MDD stands to be informative as well. Additionally, we will be able to elucidate how motor behaviors map on to stable (white matter tracts) and more temporally sensitive (functional connectivity patterns) motor network components, as well as how these networks interact. Indeed, higher-order functions linking cognition and movement take place in cortico-cortical networks, while the basal ganglia loops help to select/inhibit a particular action or sequence, and the cerebellar circuits operate in tandem to finetune these actions. These circuits work in close concert [49], and it is not possible to have a comprehensive understanding of the contributions of one circuit without examining all three.

Third, we will move beyond seed-based connectivity approaches and investigate these motor networks at rest using a graph theoretical network approach. Such a methodological approach will allow us to investigate the network dynamics of the motor network(s) as a whole, as opposed to the patterns of connectivity for one given motor seed region. Additionally, comparisons of these metrics will provide a network-level view of differences due to disease state (remitted vs. current MDD) and symptomatology (PmA vs PmR). By combining tractography and resting-state methods, we will be able to lay a foundation for the first comprehensive model of motor dysfunction in MDD, and thus provide a framework for biomarker research that will inform novel pharmaceutical and brain stimulation treatment studies.

Fourth, this study is highly innovative in its use of both actively and remitted depressed subjects, and in following these groups over 18 months. The current design will provide new, and vitally important, information on how motor behaviors and related brain networks appear in remitted and active disease states. Findings of motor disturbance (relative to controls) in remitted individuals would suggest that motor disturbance continues into remission (and perhaps reflects a vulnerability for relapse). Further, and most notably, modeling motor behavior and depressive symptom changes in these groups over time (i.e., as depressed patients improve or remitted patients relapse) will help us evaluate the potential of mechanistically relevant and easily assessed biomarkers for this populations, as well as how motor behaviors change as a function of disease course [50].

Finally, there is increasing evidence highlighting the importance of motor behaviors in a *broad range* of psychiatric disorders [13,51,52]. In recognition of this, NIMH recently added a Motor Domain to the RDoC matrix [12]. Importantly, however, no study has employed an RDoC approach to understanding these important behaviors. While the present study adopts multiple aspects of the RDoC initiative (e.g., multiple units of analysis, operationalizing PmR and PmA as continuous dimensions), it is not examining the *transdiagnostic* aspect of RDoC as it is only examining one disorder (MDD, see refs. [53,54] as exemplars of such a transdiagnostic approach). However, it should be noted that RDoC is agnostic with respect to respect to current definitions of disorders, thus, the fact that the present study is only focusing on one disorder is not inconsistent with the initiative. In sum, by employing multiple units of analysis (self- and observer-report, lab-based and ecologically valid behavioral measures, structural and functional circuitry), operationalizing PmR and PmA as continuous dimensions, and studying changes in these RDoC constructs across disease states and time, the present study will be the very first to take an RDoC approach to understanding motor phenomena in MDD.

## PRELIMINARY STUDIES

### Actigraphy in Depression

**Figure 1.**
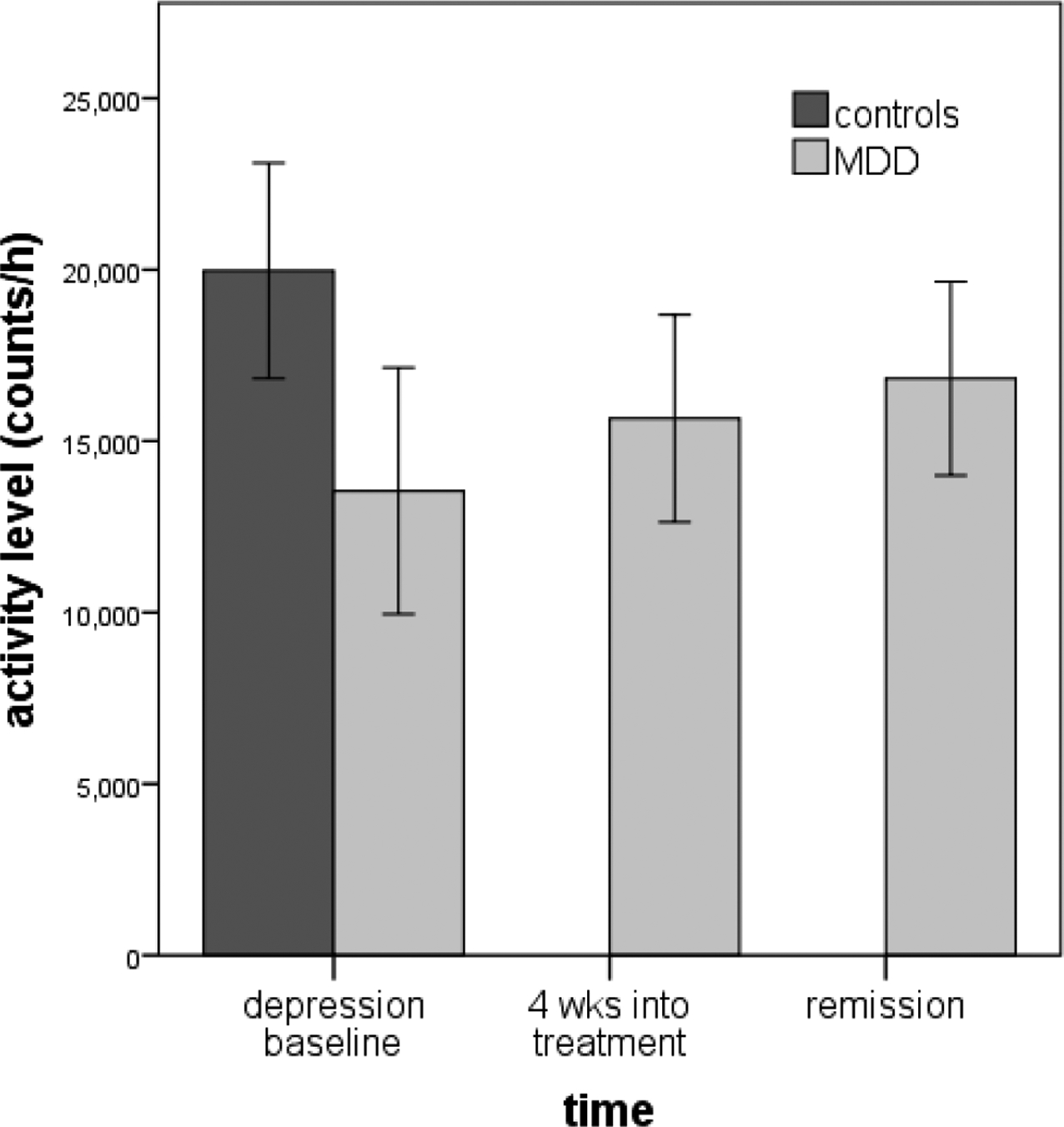
Actigraphy levels in current (*N* = 22) and remitted MDD (*N* = 11).

**Figure 2.**
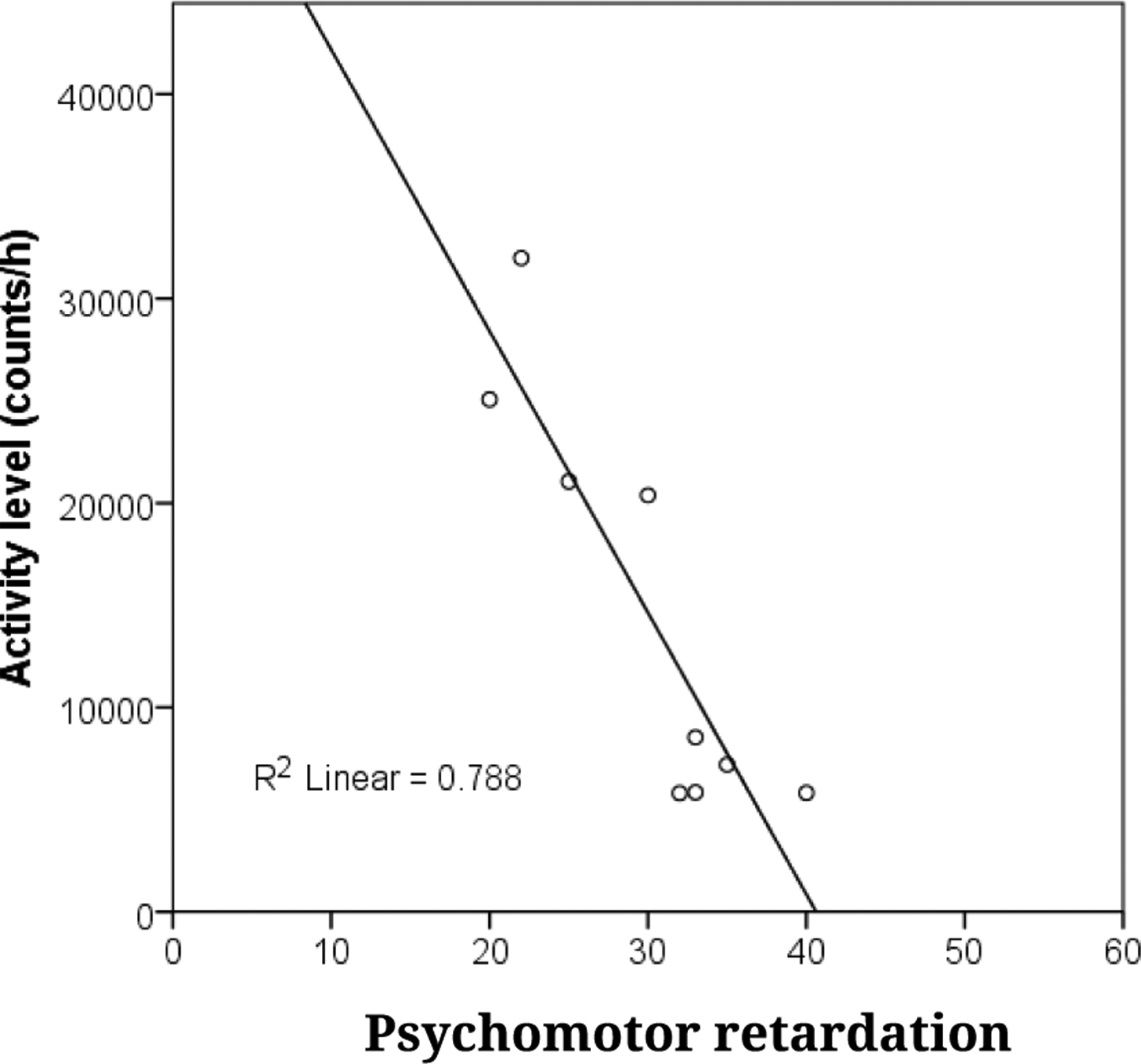
Actigraphy levels relates with Salpêtrière Retardation Scale (*N* = 9).

In support of **Aim 1**, MPI Walther showed that overall gross motor activity, as measured by wrist actigraphy, is reduced in acutely depressed individuals [27,55] and stable over 1 week [56]. This replicates other studies of MDD across the lifespan (youth, late life) [57–60]. As a preliminary test of whether this deficit continues into remission, MPI Walther examined actigraphy levels of 22 MDD patients during treatment. While activity levels increased over the course of treatment, the 11 patients who achieved remission had slightly lower activity levels than controls (Cohen’s *d* = 0.49, see Figure 1). Actigraphy-assessed activity levels also tracked with other measures of psychomotor disturbance: (a) the Hamilton depression scale item “work and activities”, even after controlling for overall Hamilton depression severity (*r* = −0.35) [56], and (b) the Salpêtrière Retardation Scale in a small pilot study (see Figure 2).

In support of **Aim 3**’s goal of examining longitudinal course, lower baseline actigraphy levels predicted a worsening of MDD symptoms over 4 weeks (*N* = 56), over and above clinician ratings of psychomotor retardation at a trend level (β = 0.23, *p* < 0.08).

### BiAffect Smartphone App

Co-I Leow conducted a pilot study on the *BiAffect* smartphone app in 24 mood disorder and healthy subjects, and found that Trail Making Test A (a well-established test of processing speed) correlated with average interkey delay (*r* = 0.50, *p* < 0.001). Additionally, independent of baseline mood [24], variability in typing behavior during a 2-week baseline period **longitudinally predicted** Hamilton depression 8 weeks later (*R*^2^ = 0.70). Typing behavior also predicted follow-up depression over and above clinician ratings of retardation and agitation at a trend (β = 0.24, *p* < 0.10). These results provide preliminary evidence supporting our use of smartphone typing behaviors as indicators of disturbance (**Aim 1**) and support the goal of examining whether PmR/PmA predicts the course of depression (**Aim 3**).

### Laboratory Motor Measures and Brain Motor Networks

#### Force variability and brain networks

**Figure 3.**
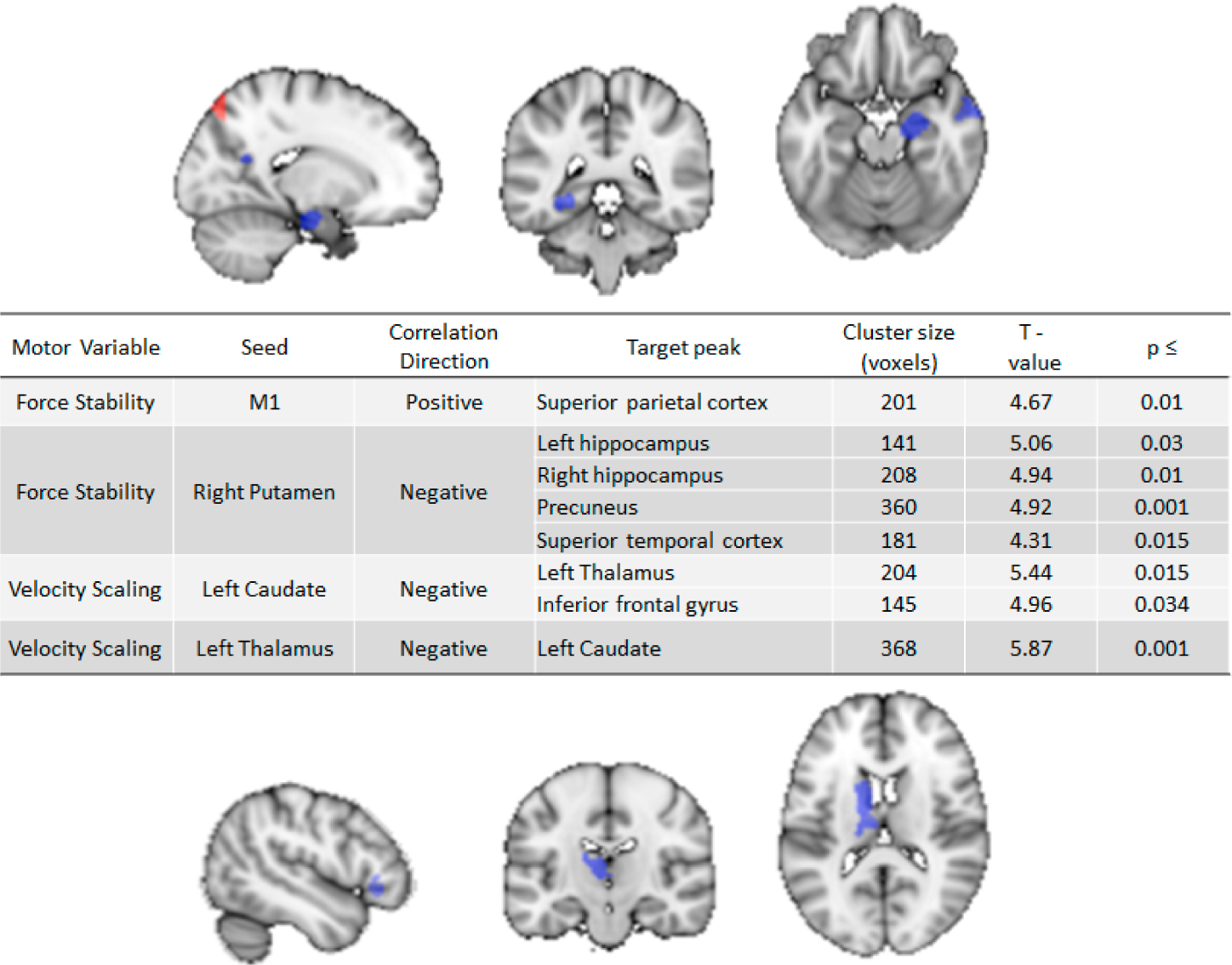
Resting state fMRI analysis showing positive (red) and negative (blue) correlations with Force Variability (top) and Velocity Scaling (bottom). (Corrected to a voxel level of *p* < 0.001 and cluster level *p*_[FDR]_ < 0.05).

In a pilot study to support the brain-motor behavior predictions in **Aim 2**, MPI Mittal evaluated the association between Force Variability (our laboratory indicator of PmA) and brain connectivity (seed-to-voxel resting state analysis) in 61 healthy controls. The seeds included motor network hubs, such as left/right caudate, left/right putamen, left/right thalamus, M1, and SMA. Increased Force Variability was related to increased connectivity between M1 and superior parietal cortex, and to decreased connectivity between right putamen and hippocampus, precuneus, and superior parietal cortex (see Figure 3).

#### Velocity scaling and brain networks

Velocity scaling, a handwriting kinematics indicator of PmR, was collected in 61 healthy controls in a pilot study examining the link between variability in slowing and brain connectivity. Increased PmR was related to reciprocal decreased connectivity between seed regions of the left caudate and thalamus. In addition, decreased connectivity between the left caudate and inferior frontal gyrus was related to increased slowing.

Taken together, these findings suggest different neural networks may contribute to PmA and PmR. However, it will be critical to evaluate this question in MDD (**Aim 2**), utilizing methods that will allow for a more comprehensive perspective of structural and functional indices of network integrity.

### Brain Correlates of Actigraphy

Further supporting **Aim 2**, MPI Walther has published numerous studies on the associations between PmR, as measured by actigraphy, and (a) whole brain resting-state perfusion and (b) white matter microstructure in major depression. Results indicated associations between actigraphy levels and (a) perfusion in the vmPFC and (b) key motor circuit regions (e.g., pre-SMA) [25,55]. Furthermore, while controls exhibited an association between actigraphy levels and perfusion within core motor areas, such as the cingulate motor area and external globus pallidus, this association was not found in MDD subjects [25]. MPI Walther has also shown that activity levels in MDD patients and controls are linked with white matter microstructure of motor pathways and limbic pathways [26,27,61,62]. However, the associations between white matter properties and activity differed between patients and controls within cortico-cortical motor pathways, suggesting that structural connectivity alterations between prefrontal and premotor/motor cortices hamper spontaneous motor behavior in patients [26]. Collectively, these findings indicate that cortico-cortical circuit alterations contribute to PmR in MDD, but contribution from the other two motor circuits is possible. Additionally, no study of MDD has investigated the associations of activity levels and functional or structural connectivity at the network level.

## RESEARCH DESIGN AND METHODS

### Overview

This project is assessing the motor functioning of 250 individuals (50 controls, 100 current MDD, 100 remitted MDD). The baseline assessment consists of (a) a diagnostic assessment (SCID) and measures of functioning, (b) laboratory (e.g., Velocity Scaling) and ecologically valid measures (e.g., actigraphy) of motor functioning, and (c) an MR scan to examine connectivity of motor circuitry. Depression/functioning and the laboratory and ecologically valid measures of motor functioning are also being re-assessed three additional times over an 18-month follow-up period.

### Diagnostic Inclusion/Exclusion Criteria

The current MDD group (*N* = 100) meets current DSM-5 criteria for MDD. The remitted MDD group (*N* = 100) meets past, but not current, criteria for MDD and have Hamilton Depression Scores (a measure of current depressive symptomatology) of less than 7. We are excluding from the remitted MDD group those with a major current DSM disorder (i.e., anxiety, trauma, substance/alcohol use disorder, obsessive compulsive-spectrum, or eating disorder). The control group (*N* = 50) does not meet lifetime criteria for any major DSM-5 disorder. However, in order to examine depressive symptoms dimensionally in the whole sample (see Data Analytic Plan and Hypothesized Results below), controls are allowed to have current subthreshold MDD. Additionally, as epidemiological studies suggest that the two MDD groups will likely be ~2/3 women [41], we are oversampling control women to ensure that the three groups have a comparable sex distribution. Given the significant lifetime comorbidity between MDD and other psychopathologies, in order to increase the generalizability of the sample, we are not excluding most *past* comorbidities from the two MDD groups. During recruitment, we are attempting to balance the two MDD groups on past comorbidities. However, individuals with lifetime ADHD or tic/Tourette’s disorders are excluded from all 3 groups given our focus on motor disturbance (note: these represent a minority of participants with MDD [63]), as well as current moderate or severe alcohol/substance use disorders. These inclusion and exclusion criteria are being determined using the SCID-5.

### Other Inclusion/Exclusion Criteria

All participants are right handed (assessed with the Edinburgh Inventory) [64] given the association between neural lateralization and handedness; able to read English; and have no serious head trauma (loss of consciousness > 2 min), neurological conditions, or personal or family history of mania or psychosis. Participants are between 18 and 60. We are excluding those older than 60 given the association between psychomotor slowing and normal aging [65], although age will be included as a covariate in our statistical models. Given the aims associated with the smartphone app, we considered requiring that participants own a smartphone as this would not limit recruitment (ownership rate in this age range is ~92%) [20] and providing a phone to those who never owned one before might yield a subsample with different typing behavior. However, this might bias our sample to be more affluent, so this is not an inclusion criterion (although we will include “previous smartphone ownership” as a covariate in our models).

### Medication

The role of medications is an important consideration in our study. A blanket exclusion would not be feasible or scientifically justified given that (a) PmR and PmA are rare side-effects for antidepressants (ATD) [66], (b) exclusion would make recruitment difficult as many eligible subjects will be taking ATDs, and, most importantly, (c) this strategy would yield a non-generalizable sample. Instead, we are choosing to exclude subjects with long-term recent exposure to specific compounds that are most likely to impact motor function. Specifically, subjects on continuously administered medications that impact dopaminergic (DA) functioning (e.g., immunomodulators, anticonvulsants, and ATD (such as bupropion and nortriptyline)) are being excluded given the potential impact of DA, a neurotransmitter important to motor functioning [67–69]. Subjects periodically taking medications that impact dopamine or other compounds that may impact motor functioning (e.g., antibiotics, antihistaminic, or antiemetic) will undergo a withholding period of four weeks prior to assessments. However, we are only excluding those who use benzodiazepines (BZO) 3 or more times per week as 7–10% of MDD patients take BZO [70] (and thus exclusion would decrease generalizability) and a power analysis excluding 7–10% of the current and remitted MDD groups did not decrease power below 80% (allowing us to run analyses with and without those on BZOs). We will thoroughly note subjects’ dosages and types of medications (for all classes, including BZOs), and examine whether medication status and/or class should be included as covariates in our model.

### Feasibility of Recruitment

We are posting advertisements on websites and at clinics, and are attempting to recruit subjects from MPI Shankman’s recently completed MDD study (R01 MH098093). We will enroll 5.31 subjects each month during the 54-month recruitment period, a rate consistent with prior studies by MPI Shankman.

### Attrition

Our recruitment goals and power estimates account for attrition and data loss. First, for Aims 1 & 2, we conservatively estimate (based on our prior work) that 15% of subjects will be excluded due to neuroimaging motion and/or general data loss. Thus, 287 subjects will be recruited to yield an *N* of 250. Second, we powered Aim 3 to have an *N* of 180–70 current MDD, 70 remitted MDD, and 40 controls. This sample is smaller because (a) growth curve modeling with 3 follow-ups allows for a smaller *N*, (b) subjects recruited after month 6 of year 4 will not have completed their 18-month follow-up by the end of year 5, and (c) general feasibility concerns about conducting 3 follow-ups on a larger sample (although each follow-up assessment is <2 h). This reduced targeted *N* will allow us to naturally lose 28% of subjects to attrition during the follow-up.

## STUDY INSTRUMENTS

A key aspect of NIMH’s RDoC initiative is to utilize multiple units of analysis to measure a construct [40,71,72]. The current study adopts this strategy by measuring PmR and PmA via self-report (IDS), interviewer’s observations (MARS/CORE), and multiple behavioral measures (Velocity Scaling [PmR], Force Variability [PmA], Actigraphy, and smartphone typing behavior). Each measure is described below.

### Baseline Ratings of Psychopathology and PmR and PmA

At baseline, psychiatric diagnoses are being assessed with the SCID-5. To determine inter-rater reliability, a 2nd interviewer will re-score 10% of videotaped SCIDs annually. We will also assess depression with the Hamilton Depression Scale (interview) [73] and the Inventory for Depressive Symptoms (self-report; IDS) [74] for secondary analyses examining depression severity dimensionally. Measures of social and occupational functioning (SOFAS) [37] and overall quality of life (World Health Organization’s Quality of Life [WHO-QOL]) [38] will also be administered.

Observer ratings of PmR and PmA will be made from videos of the SCID interviews. Raters will use the CORE, the gold standard measure of PmR and PmA, and the Motor Agitation and Retardation Scale (MARS), an inventory that, unlike the CORE, only assesses the motor components of PmR and PmA (and not the cognitive components, e.g., inattentiveness). These observer ratings will not be made by the SCIDer so that PmR/PmA ratings can be blind from diagnosis.

### Laboratory Assessments of Motor Disturbance

#### Lab measurement of PmA

Laboratory PmA is being assessed using Force Variability, a validated measure of PmA in MDD [75]. Subjects are asked to match a target on a monitor by applying constant pressure on a strain gauge with their index finger as steadily as possible (9 trials of varying force for each hand (approximately 350 cN of force per trial)). The variability in the subject’s applied force reflects the subject’s dyskinesia and is the direct result of irregular muscle contractions that produce changes in measurable force over time. The task consists of three 20-s trials, separated by 5-s rest periods. After removing any tremor component (via a low pass filter), the segment with the greater range in force (i.e., the force minima and maxima over the medial 80% of each segment) will be subjected to quantitative analysis of error. Force Variability is defined as the coefficient of variation from the mean and standard deviation of the force waveform.

#### Lab measurement of PmR

**Figure 4.**
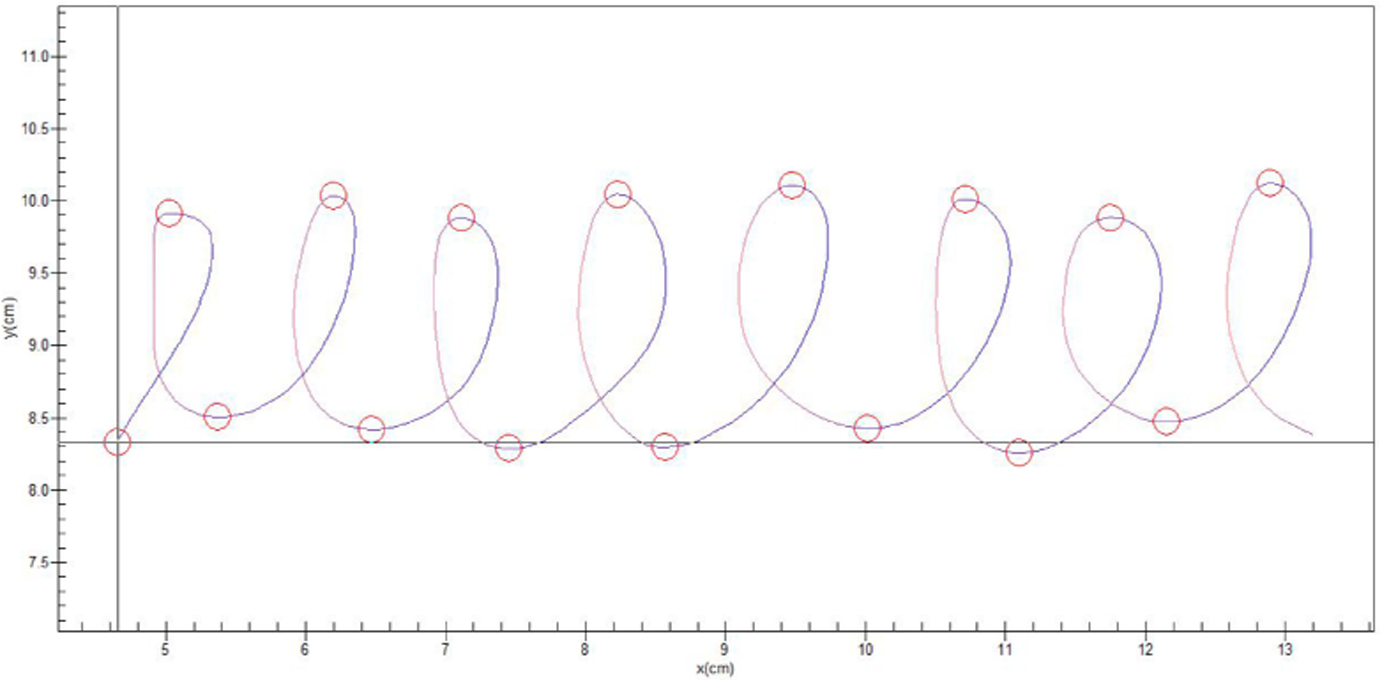
Loops from a 4 cm trial. These will be compared with those from a 2 cm trial to calculate a VS ratio (an indicator of PmR).

Handwriting samples are being obtained to compute Velocity Scaling to index PmR [76]. Lower values of Velocity Scaling reflect the slowing or inability to increase pen movement velocity across shorter and longer target distances, and have been linked to motor slowing in several clinical populations [17,18,77]. Handwriting samples are acquired using Neuroscript MovAlyzeR software (http://www.neuroscript.net), installed on a Fujitsu T901 tablet computer, and a non-inking pen. Subjects are instructed to write 8 loops continuously for 3 trials within a 2 cm or 4 cm vertical boundary, using their dominant hand (see Figure 4). Subjects are instructed to write at their normal speed. Each trial consists of 16 vertical strokes, which will be segmented and processed for target variables: peak vertical velocity and absolute size of stroke. Valid trials will include at least 10 segments. A regression with peak vertical velocity as the predicted variable will be entered, with absolute size of the stroke (to account for variance of individual stroke sizes) and condition (2 cm and 4 cm) as covariates [78].

#### Postural control

As an additional laboratory measure of motor functioning, we are also assessing individuals’ postural control using an instrumental balance task, a putative probe of cerebellar abnormalities [79]. Data is acquired using an Advanced Mechanical Technology Incorporated (AMTI) AccuSway force platform (Watertown, MA, USA). Participants are instructed to stand still on the force platform with their arms rested at their sides, look straight ahead, and complete four 2-min-long task conditions: (1) feet together with no cognitive load, (2) feet together with cognitive load, (3) feet shoulder-width apart with no cognitive load, and (4) feet shoulder-width apart with cognitive load. Cognitive load is manipulated by having participants count backwards by 13 from 1000. The center of pressure (COP) is the variable of interest, and it is recorded at a sampling rate of 50 Hz. We will apply a 9th-order Butterworth filter with a 20-Hz cut-off frequency to isolate the low-frequency postural sway process in the recorded data. Higher COP area is associated with poorer postural control.

### Ecologically Valid Measures of Motor Disturbance

#### Actigraphy assessment

Wrist actigraphs (Philips Respironics, USA) are placed on the nondominant arm for continuous recording of motor activity for 1 week (at 30 s intervals). Actigraphy uses accelerometers to collect movement counts per interval. Data will be processed in Dr. Walther’s lab, focusing on activity levels (i.e., average activity counts per hour during wake periods of the day). Subjects also complete a sleep log to indicate wake periods and for cross-validation [56]. Time series analysis will be used to test for temporal stability of the movement patterns [75]. Specifically, the time series of logged activity data will be subject to a partial autocorrelation function (PACF) on defined actigraphy data periods of several hours when most subjects are awake (e.g., from 9:00 AM to 12:00 AM). The PACF focuses on the time series of movement counts in chronological order and indicates the number of lags with significant autocorrelation (with the correlation in the opposite direction partialled out). In time series with low numbers of significant lags (e.g., 2), the movement count in lag 1 is only associated with movement in lag 2. Higher numbers of significant lags are interpreted as more stable movement patterns. The PACF derived number of lags is covaried for the total amount of movement. PmR will be defined as the amount of total activity level, and PmA will be defined as unstable movement patterns (i.e., low number of significant lags). As an exploratory measure, actigraphy data will also be used to analyze circadian variation of motor activity [80].

#### BiAffect smartphone app

BiAffect was developed by Co-I Leow and colleagues and won the RWJ Foundation’s Mood Challenge award in 2017. BiAffect tracks metadata of character category (e.g., alphanumeric, lower vs upper case), timestamps of keypresses, distances between consecutive keypresses, backspaces, autocorrect, and typos. BiAffect runs on all smartphone operating systems, utilizes an open source framework, and has a HIPAA compliant data management system (Safe Bionetworks). The indicator of PmR is the average interkey delay over 2 weeks, and PmA is indicated by daily variability in typing speed. It should be noted that these classifications are made tentatively as PmA may lead to difficulty typing (leading to a higher interkey delay). We will also explore other variables from the Biaffect app (e.g., Backspace Ratio [# of backspaces/total keypresses] and Autocorrect Ratio [# of autocorrect events/total keypresses]).

### Neuroimaging Assessment

#### Data acquisition

All scanning is being completed using a 3 tesla Siemens MAGNETOM Prisma scanner. The scanner has a full set of established sequences for functional, anatomic, and diffusion weighted imaging, and is also equipped with automated shimming. We use foam padding to minimize head motion, though we will correct for any motion during preprocessing. The study employs sequences from the Human Connectome Project (HCP) Lifespan protocol. There are several benefits to this protocol. First, these are publicly available, well-validated protocols and will aid in future reproducibility and data-sharing efforts. Second, the sequences are relatively short, which is optimal for the clinical population being studied here (total time for all scanning is 40 min). Furthermore, the imaging methodologies employed here eliminate potential task confounds and show a high degree of reproducibility within subjects and across testing sites [81,82].

Our specific parameters are outlined in detail on the HCP website (protocols.humanconnectome.org). We are collecting a high resolution T1-weighted anatomical image with whole-brain coverage (MPRAGE; 0.8 mm^3^ isomorphic voxels, 208 interleaved slices; FOV = 256 mm) to facilitate normalization for our resting state and diffusion images. Resting state functional blood oxygen level dependent (BOLD) connectivity will be collected in two separate multi-band imaging scans, each lasting 5:12 min, with complete brain coverage and 2.0 mm^3^ isomorphic voxels, and with opposite phase encoding directions (anterior to posterior, and posterior to anterior). The two shorter scans will help to minimize movement confounds during data acquisition. Finally, all participants will undergo 4 diffusion weighted imaging scans with the following parameters: two with 98 gradient directions and two with 99 gradient directions. Each scan has 1.5 mm^3^ isomorphic voxels and two shells, β-value = 1500 s/mm^2^ and 3000 s/mm^2^ interleaved at a 1:2 ratio, 6 β0 images, and will last 5:38 min. Each couplet of diffusion scans is collected with opposite encoding directions. The additional diffusion scans are necessary for modelling crossing fibers using tractography in our proposed analyses.

#### Resting state connectivity processing

**Figure 5.**
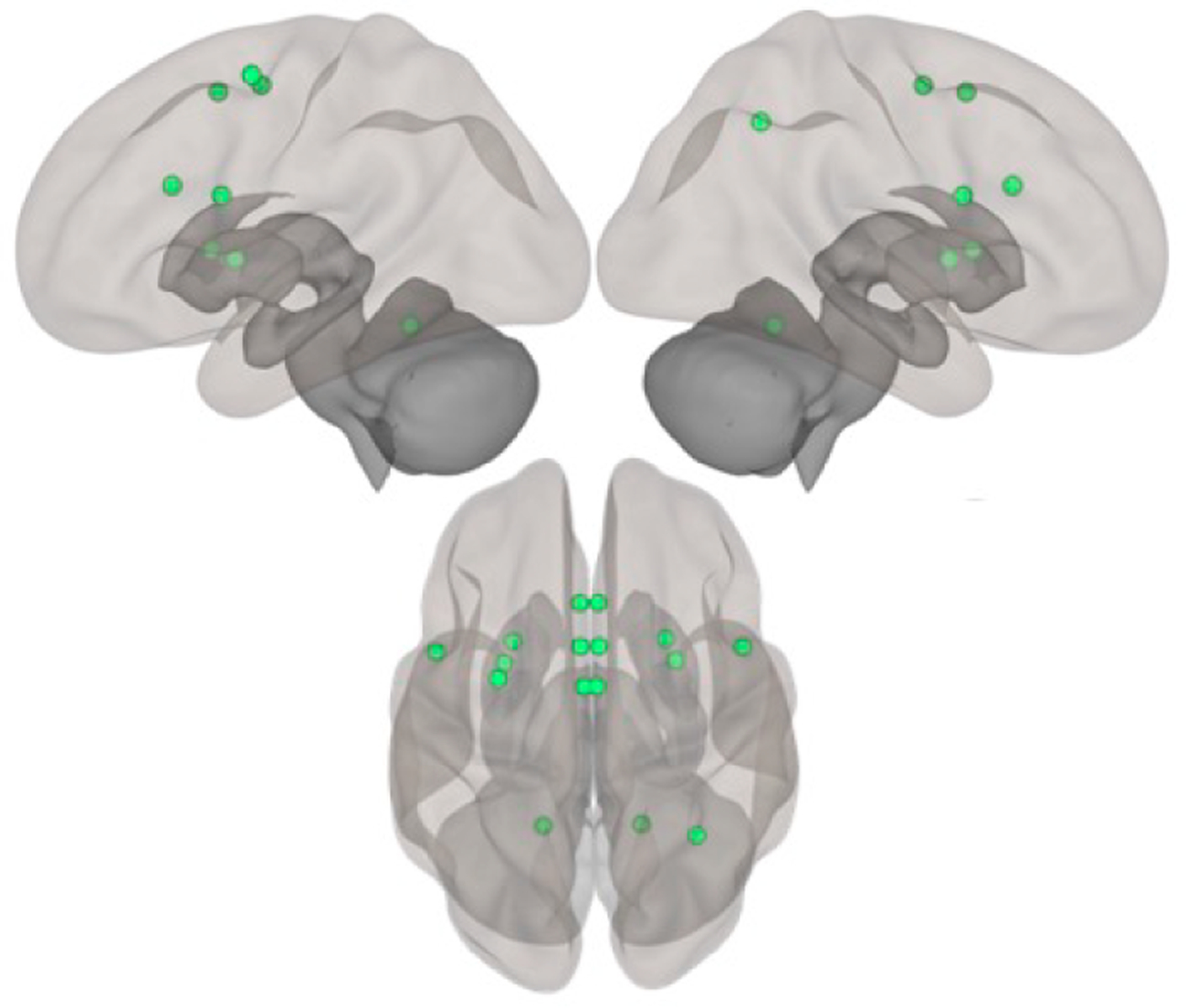
Regions in the motor network to be used for graph theory analysis. Top = left & right hemispheres; Bottom = superior view.

We will use the HCP processing pipeline [83] to investigate the connectivity of these circuits. We will take a graph-theoretical approach, implemented using the Brain Connectivity Toolbox [84] (https://sites.google.com/site/bctnet/). We will also investigate the network-based statistic (NBS) [85]. The NBS allows us to investigate overall network dynamics and is particularly informative when looking at differences across diagnoses [86] (and, as such, stands to be particularly informative for our investigation here). For the motor networks, we will create regions-of-interest (ROIs) for the key nodes of the motor circuits [13], defined *a priori*. We will use spherical seeds, 6 mm in diameter. These nodes will be put together into one large motor network, concatenating across the circuits. The network nodes to be used in our analyses are pictured using the Brain Connectivity Toolbox (see Figure 5). We will quantify the clustering coefficient (C) and the clustering strength (S), in addition to the NBS. C measures the degree of local connectivity within a network, while S quantifies how closely network nodes are connected. This will allow us to not only assess the small-worldness of the motor network, but to also look at overall network dynamics. In addition, we will investigate these metrics in the three motor circuits discussed [13]. We will then compare the organization and graph metrics between the groups. We will also compute the associations between the graph measures and the measures of PmR and PmA and psychiatric symptomatology.

#### Diffusion imaging processing

DTI data will be analyzed using a probabilistic tractography approach to target tracts of interest, connecting key motor nodes of the brain. We will follow similar methodologies employed in our recent work on cerebellar circuits and thalamo-hippocampal connections [49,87,88]. Diffusion data processing will be completed using FSL’s FDT toolbox. We will correct for motion and eddy current distortion. We will then use BEDPOSTX to calculate diffusion parameters at each voxel [89]. We will compute probabilistic tractography analyses for several tracts of interest in FSL using ProbtrackX. We will model the specific parameters after our prior work [49,87,88]. In order to test our hypotheses related to motor disturbance in MDD, we will look at several tracts tapping into the striato-cortical, cortico-cortical, and cerebello-thalamo cortical circuits. These tracts include the connection from cerebellar lobule V to the thalamus, from the thalamus to M1, the striato-cortical connections from the putamen to both M1 and SMA/pre-SMA, and caudate to pre-motor cortex [90], and, finally, the cortico-cortical connections between DLPFC and SMA. We will compute the probabilistic tractography between the two regions of interest, which will serve as endpoints. From these tracts, we will compute fractional anisotropy (FA), radial diffusivity (RD), and axial diffusivity (AD).

### Longitudinal Follow-up Assessment (for Aim 3)

Psychopathology (SCID, symptom measures, and functioning) and motor behaviors (Force Variability, Velocity Scaling, 2 weeks of BiAffect App data, and 1 week of actigraphy) are being re-assessed at 6, 12 and 18 month follow-ups. At each follow-up, we are also administering the Longitudinal Interview Follow-up Evaluation (LIFE) to assess monthly psychopathology since the previous assessment. The LIFE is critical as it provides a more nuanced assessment of the timing and duration of psychopathology episodes that are not assessed in the SCID. As discussed in the Attrition section above, we plan to only follow-up with 70 current MDD, 70 remitted MDD, and 40 controls.

#### Reproducibility and Rigor

The current study has taken a number of steps to ensure reproducibility and rigor: (1) employing reliable and valid measures that have been used in prior studies of motor disturbance [75,76,91]; (2) ensuring sufficient power to test study aims; (3) creating a detailed statistical plan; (4) utilizing a representative, community sample that will likely have adequate variability in our measures; (5) detailing a plan for appropriate handling of missing data (see Data Analytic Plan and Hypothesized Results below); (6) facilitating reproducibility of results by other research groups, as all de-identified data will be placed in NIMH’s RDoC database (see Resource Sharing Plan); and (7) employing imaging methodologies (fcMRI, DTI) that have a high degree of within subject test-retest reliability and are highly replicable across scanners and labs [81,82,92].

### Data Analytic Plan and Hypothesized Results

Data collection began in the fall of 2019. The following data analytic plan outlines how we will conduct our analyses to test the study’s aims. Analyses for outliers, non-normal distributions, and nonlinear relations will be conducted; data transformations will be considered where appropriate. Missing data will be accommodated using robust maximum likelihood estimation procedures or multiple imputation, as recommended by modern missing data guidelines [93]. Preliminary analyses will examine whether any of the following covariates should be included: years of education, ethnicity/race, medication status (including specific classes; see Research Design and Methods above), and whether the subject had a smartphone vs was loaned one for the study. We will also explore age and variables related to prior course (age of onset, number of episodes, etc.) as potential moderators given their potential effects on motor behavior. Conclusions for all aims will primarily be determined based on effect sizes with 95% confidence intervals, rather than statistical significance, in order to maximize reproducibility of our findings.

#### Sex differences.

As there are important sex differences in the presentation and risk factors for MDD (see Sex Differences in Motor Disturbance), we will also test for sex differences by including sex as a moderator in the below statistical models.

##### Aim 1—Compare three groups on laboratory and ecologically valid measures of PmR and PmA.

To test this aim, we will conduct separate MANCOVAS for each indicator of PmR and PmA with group (current MDD, remitted MDD, and controls) as a between groups factor and covariates identified in our preliminary analyses. These will be followed-up with simple effects to test **Hyp. 1a and 1b** regarding which groups differ from each other. Consistent with the RDoC initiative, we will examine multiple indicators of PmR and PmA:

- PmR: Velocity Scaling ratio (lab behavior), lower actigraphic activity and slower typing speed on smartphone (outside of lab behavior), IDS (self-report (item 23)), and CORE/MARS (interviewer-report).
- PmA: Force Variability (lab behavior), variability in actigraphic activity and typing speed (outside of lab behavior), IDS (self-report (item 24)), and CORE/MARS (interviewer-report).

#### Exploratory analyses.

As an exploratory aim, to reduce the number of PmR/PmA indicators, we will also conduct a confirmatory factor analysis to create PmR and PmA latent variables (see ref. [94] for a similar RDoC approach). These latent factors might not account for a large portion of the indicators’ shared variance due to the high method variance (behavior, self-report, etc.). However, the latent factors will reflect the core of PmR and PmA. Factor scores for PmR and PmA will then be used as dependent variables in the above ANCOVAs.

#### Aim 1 power.

We used G × Power Software [95] to compute the power estimates from an ANCOVA test with 3 groups and 3 covariates. Using established [96,97] guidelines and methods to calculate the required sample sizes, the targeted sample of 250 (which takes into account 15% attrition) will have greater than 80% power to detect medium effect sizes (*f* = 0.25) at α = 0.05. Thus, the analyses for aim 1 are adequately powered.

##### Aim 2. Examine the neural mechanisms (structural (white matter) and functional (resting state fMRI) connectivity) of PmR/PmA in MDD.

As discussed in Possible Neural Mechanisms of PmA and PmR above, given the lack of foundational studies in this area to draw from, we have elected an innovative, but still primarily conservative, test of the neural mechanisms underlying motor disturbance in MDD (i.e., examining the structural and functional connectivity of the 3 primary brain circuits that have been shown in human and animal studies to regulate motor behaviors [13]). With this broad approach and accompanying strategy for understanding network coherence within and across the circuits, as well as the large sample size and optimized statistical strategy, our study is well-powered to detect small to medium effects that will help us to isolate which brain mechanisms are implicated in motor symptoms in depression. Our hope is that our findings will provide a sound foundation for future studies to build upon.

- Hyp 2a: The motor system circuitry of the three groups will exhibit different structural and functional connectivity (specifically, in a cortico-cortical motor network and basal ganglia mediated motor circuitry).
- Hyp 2b: Lab and ecologically valid measures of PmR/PmA will correlate with neural circuitry abnormalities.

We will focus our analysis on a model of the motor network made up of the three circuits discussed [13]. In addition, for the resting fMRI data, we will investigate graph theory measures of these three motor circuits as these metrics are predicted to relate differently with PmR and PmA. We will compare the organization and graph metrics between the groups. To test **Hyp. 2a** with respect to *functional connectivity*, we will use group × graph metric (3 × 3) mixed model ANOVAs for the network measures in question. This analysis will allow us to test the hypothesis that network dynamics are differentially impacted across motor circuits in those with MDD, relative to individuals with remitted MDD and healthy controls.

To test **Hyp. 2a** as it relates to *structural connectivity* of motor circuits, we will conduct a mixed model group (current MDD vs remitted MDD vs controls) by motor tract (3 × 6) ANOVA for each white matter measure (FA, RD, AD). The motor tracts are the cerebello-thalamic connection, the thalamo-M1 white matter tract, cortico-striatal white matter connections between the putamen and M1 and SMA/pre-SMA, respectively, and caudate to pre-motor cortex, and finally, cortico-cortical white matter connecting DLPFC and SMA. These analyses will be Bonferroni corrected, given the multiple comparisons across white matter measures.

**Hyp. 2b** will be tested with multiple regression models using measures of network coherence, and with white matter metrics as dependent variables and the indicators of PmR/PmA as independent variables (and relevant covariates as described above). We will also explore whether group (current vs remitted vs control (dummy coded)) moderates the association between PmR/PmA and the neural measures. We hypothesize (albeit tentatively) that PmR will be associated with reduced structural connectivity and less global efficiency of resting state parameters (from graph theory metrics) of cortico-cortical circuits. PmA is expected to relate to increased functional and structural connectivity in the cortico-basal ganglia loop. Given the novel nature of this aim, we will also explore other PmA/PmR and motor circuit associations as well as interactions of the 3 circuits.

Exploratory analyses will examine whether the network connectivity measures longitudinally predict the course of depressive symptoms and motor disturbance. These analyses have the potential to elucidate the predictive utility of motor neurocircuitry on disease trajectories over time.

#### Aim 2 power.

The primary analyses for Aim 2 rely upon ANCOVA with three groups and three covariates. Thus, the power considerations for Aim 2 are comparable to that of Aim 1. Notably, however, we are employing multimodal neuroimaging measures, in which issues related to statistical power are notoriously complex [98]. The large sample size employed here is substantially larger than what is often used in this type of research. Further, our analysis plan avoids many of the challenges and pitfalls associated with more traditional resting state connectivity, or whole brain diffusion tensor imaging analyses. Traditional seed-based analyses of resting state data, or whole-brain diffusion tensor imaging analyses, are subject to concerns regarding multiple comparisons as modelling is conducted across every voxel in the brain, which also presents unique challenges with respect to statistical power. With our *a priori* data-driven approach using graph theory, we are better powered to detect group differences in these network parameters, and we are less susceptible to the issues of false positives that can occur, even with statistical corrections in whole brain analyses.

##### Aim 3—Re-assess PmR and PmA and depression/functioning three times over 18-month follow-up period.

The analyses for this aim will use multilevel modeling (MLM) as the repeated observations (e.g., follow-up at 6 months, 12 months, etc.) will be “nested” within participants. This approach is superior to repeated measures ANOVAs as it (a) allows participants to have missing observations (as it uses maximum likelihood procedures to estimate parameters), (b) has less stringent overall assumptions, and (c) accounts for individual differences in baseline responses (random intercept) and changes over time (slopes) [99,100].

Equation 1 presents an example of the multi-level model to test **Hyp. 3A** that baseline measures of PmR and PmA will predict a worse depression/functioning course over time. We will consider the model for a continuous outcome of depression denoted as Hamilton_*i*,*t*_, recorded at time point *t* on subject *i*. Similar models will use functioning as the outcome. The multi-level model is given as follows:

(1) $${\text{Hamilton}}_{i,t}=\beta_{0}+{\text{TIME}}_{i,t}\times \beta_{1}+{\left(\text{PmR}/\text{PmA}\right)}_{i,1}\times \beta_{2}+{\left(\text{TIME}\right)}_{i,t}\times {\left(\text{PmR}/\text{PmA}\right)}_{i,1}\times \beta_{3}+\varepsilon_{i,t}$$

where *β*_0_, *β*_1_, *β*_2_ and *β*_3_ are the fixed effects, and ε_*i*,*t*_ is the residual error, which will be assumed to have a covariance matrix that models the autocorrelation among repeated observations.

Hypothesis testing will test for significant interactions between (TIME) and baseline (PmR/PmA)_*i*,1_. Specifically, a significant positive (negative) regression coefficient for the vector *β*_3_ indicates that baseline measures of PmR and PmA predict a worse depression/functioning course over time. We will also explore whether group moderates these associations (i.e., whether the effect of PmR/PmA on the course of MDD is different for each of the 3 groups) by adding a group-level random effect (dummy coded) to the model in Equation 1, along with the interaction of group × PmR/PmA. Analyses will be conducted in *R* (lme4 package).

To test **Hyp. 3b** that over time, changes in overall depressive symptoms will relate to changes in PmR and PmA, a multilevel model will be conducted with the following equation.

$${\text{Hamilton}}_{i,t}=\beta_{0}+{\text{TIME}}_{i,t}\times \beta_{1}+{\left(\text{TIME}\right)}_{i,t}\times {\left(\text{PmR}/\text{PmA}\right)}_{i,t-1}\times \beta_{2}+\varepsilon_{i,t}$$

The significance of *β*_2_ will be used to test whether previous PmR or PmA scores (i.e., time = 1) predict the subsequent Hamilton score (after adjusting for previous Hamilton score). The opposite directional model (i.e., depression → subsequent motor disturbance) will also be run to test the specificity of the motor → depression pathway. As with the Hyp. 3a models, we will also explore whether group moderates these associations. Comparable models will be run with functioning.

Models for Hyp. 3a and 3b will also be run using (a) Hamilton scores calculated without the Hamilton’s PmR and PmA items (item #8 & 9), to rule out criterion contamination (i.e., where the criterion variable contains the predictor variable), and (b) Psychiatric Status Ratings from the depression module from the LIFE.

#### Aim 3 power.

Power analysis and sample size calculations for linear mixed effects models are challenging because assumptions have to be made about many key model parameters. Note, however, that linear mixed effects model can handle missing values, and thus generally produce larger statistical power than a repeated measures ANOVA analysis with the same sample size. Therefore, we used G*Power Software to compute conservative power estimates from a repeated measure within-between interaction ANOVA. To ease the computation for power analysis, we followed [101] to approximate the distribution of the continuous variables PmA and PmR using discretization with 20 equal-size bins for power analysis. Using an alpha of 0.05, and assuming an intraclass correlation of 0.5 between the repeated measures from the same participant, the sample size of 180 that will participate in the follow-up will have more than 80% power to detect medium effects (*f* = 0.25; note: as stated above, this *N* factors in the planned attrition from the larger sample of 250).

#### Incremental validity of behavioral measures

For each aim, we will also examine the incremental validity of the behavioral measures (e.g., Velocity Scaling, Actigraphy) over and above traditional diagnostic measures of psychomotor disturbance (e.g., CORE, self-report) by including both types of indicators of PmR (or PmA) in the same model. These analyses are designed to test whether the more fine-grained behavioral measures of PmR/PmA contribute additional predictive power over the coarser diagnostic measures.

#### Exploratory analysis: Depression as a dimension

As several studies suggest that MDD might be better conceptualized dimensionally rather than categorically [102,103], in the three aims, we will also explore the impact of depression when it is defined as a dimension instead of a category. These analyses will use an average of Hamilton and IDS depression severity (i.e., an interviewer- and self-report depression measure), instead of group (current vs remitted MDD vs control), in the above models. As these analyses employ fewer degrees of freedom than the three group variable, they will have more statistical power to detect effects.

### Potential Issues, Alternative Approaches, and Future Directions

It is important to note that, while our study focuses on abnormal motor behaviors, our neuroimaging methods are sensitive to motion. However, in our prior studies [48,82], as well as other studies of depression [104] and disorders associated with movement dysfunction (e.g., Huntington’s disease [105]), investigators have effectively employed tactics to limit motion in the scanner and control for motion effects analytically. To address this issue, however, we are attempting to limit motion during the scan session (e.g., foam cushioning to fix head in place) and employ state-of-the-art methods to account for the motion artifacts in our analyses.

When completed, the current study will map out motor dysfunction in MDD for the first time using state-of-the-art methods. Our “RDoC approach” of employing multiple indicators of PmR and PmA will allow for an in-depth investigation of these behaviors, and the use of smartphone technology, in particular, will help bridge our results to future real-world translational applications. Further, because the motor circuits are well understood, but have not been extensively studied in MDD, our multimodal imaging approach to determining pathophysiology will serve as the basis for a novel theory of motor dysfunction in depression. Even if our imaging hypotheses are not supported, we could explore other important questions (e.g., whether the course of depression is linked to specific neural seeds at baseline). Similarly, if remitted MDD subjects do not differ from controls (i.e., Hypothesis 1b is false), then motor disturbance could be a state, rather than trait, marker of MDD. In this case, we could still test the state effects of motor disturbance in hypothesis 3a (whether PmR/PmA predict a poorer course over 1.5 years) and 3b (changes in depression relate to changes in motor disturbance).

In short, because motor disturbance is a central feature of MDD and so little work has been done in this domain, this is a low risk, high reward project. To maximize the potential for success, we put together an expert team of investigators, a comprehensive approach to studying phenotype and mechanism, and a well-powered conservative analytic strategy. The resulting project, therefore, has significant potential to identify novel treatment targets, and test promising biomarkers that will ultimately improve detection, monitoring, and treatment of a devastating and prevalent psychiatric disorder.
